# Diffuse Large B‐Cell Lymphoma Combined With Paroxysmal Nocturnal Hemoglobinuria: A Rare Case Report

**DOI:** 10.1002/ccr3.71394

**Published:** 2025-11-19

**Authors:** Zhen zhen Liu, Da lin Di, An hua Feng, Jie Yu, Hai ying Wang, Lili Qin

**Affiliations:** ^1^ Department of Hematology Affiliated Hospital of Shandong Second Medical University Weifang China; ^2^ Department of Immunology Shandong Second Medical University Weifang China

**Keywords:** diffuse large B‐cell lymphoma, hemolysis, paroxysmal nocturnal hemoglobinuria, venous thrombosis

## Abstract

Diffuse large B‐cell lymphoma (DLBCL) is a common hematological malignancy, and some patients may present with autoimmune hemolytic anemia (AIHA). However, the coexistence of DLBCL and paroxysmal nocturnal hemoglobinuria (PNH) is rare. PNH patients may develop myelodysplastic syndrome (MDS) or acute myeloid leukemia (AML), but lymphomas are rarely seen. Here, we focus on a 46‐year‐old female patient afflicted with PNH for 3 years before being diagnosed with DLBCL. Different from DLBCL accompanied by AIHA, this case did not benefit from the R‐CHOP regimen. This case demonstrates that PNH can coexist with malignant lymphoma, which is a rare finding. It also enhances our understanding of the clinical management of DLBCL coexisting with PNH.

**Summary**

- DLBCL coexisting with PNH is very rare and has distinct clinical features from AIHA.
- Comprehensive evaluation of pathology, clinical stage, PNH clone proportion, and treatment response is crucial before chemotherapy.
- Close monitoring of blood parameters, liver/kidney function, LDH levels, and prophylactic anticoagulation can improve chemotherapy tolerance and outcomes.

## 1 Introduction

PNH caused by the mutation in the phosphatidylinositol glycan class A (PIG‐A) gene, manifesting clinically as complement‐mediated hemolysis due to the deficiency of glycosylphosphatidylinositol‐anchored proteins (GPI‐Aps) on blood cells [1]. Some PNH patients may be at high risk of developing myelodysplastic syndrome (MDS) or acute myeloid leukemia (AML) [2]. Complement inhibition, such as eculizumab and ravulizumab, is effective in treating PNH [3]. DLBCL is an aggressive malignant tumor and the most common type of lymphoma worldwide with an initial manifestation of rapid growth of one or more lymph nodes [4]. Some DLBCL patients who were prone accompanied by hemolytic diseases may achieve CR with the R‐CHOP regimen. Notably, DLBCL in combination with PNH is rarely reported [5, 6]. Here, we present a case of a 46‐year‐old female PNH patient with DLBCL who died from lung infection, hemolysis, and multiple vein thrombosis after two courses of R‐CHOP chemotherapy despite an initial partial therapeutic response. This case report retrospectively analyzes the relevant literature and the patient's treatment course to elucidate the potential underlying mechanisms associated with PNH and DLBCL while highlighting the challenges and prognostic implications that may arise in such cases.

## 2 Case History/Examination

The patient is a 41‐year‐old female with a history of hemolytic anemia diagnosed in other hospitals for 3 years and was treated with intermittent oral glucocorticoids and blood transfusions. One year before admission, the patient had new‐onset right lower limb pain accompanied by decreased muscle strength and impaired mobility. Over the past 4 months, she had experienced a weight loss of approximately 10 kg. About 10 days before presenting to our hospital, computed tomography (CT) scans of the chest, abdomen, and pelvic cavity performed at other facilities revealed multiple enlarged lymph nodes in the left clavicular region, retroperitoneum, and pelvic cavity. Fluorescently labeled inactivative aerolysin (FLAER) cytometry revealed an absence of GPI‐APs on 79.14% of granulocytes, and 73.85% of monocytes. Physical examination revealed a pale face, jaundiced skin and sclera, enlarged lymph nodes in both the cervical and axillary regions, and Grade 2 muscle strength in the right lower limb.

## 3 Investigations and Treatment

**FIGURE 1:**
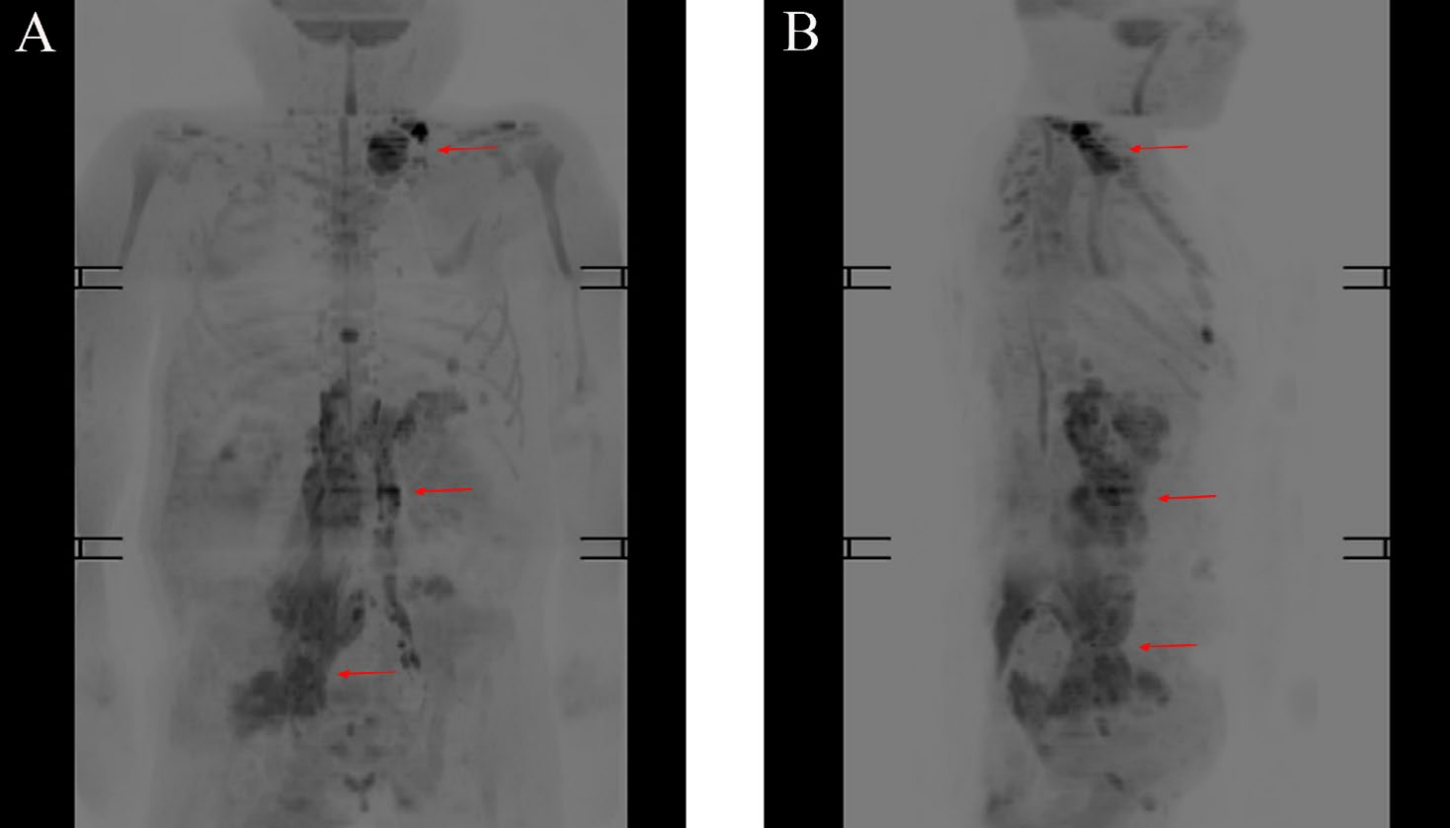
Whole‐body magnetic resonance imaging (MRI) with diffusion‐weighted imaging (DWI) shows multiple enlarged lymph nodes (arrows) in the left neck base, left upper and lower clavicle regions, abdominal cavity, retroperitoneum, and right pelvic wall. It indicated that lymphoma had invaded the stomach, bladder, right gluteus maximus, and lumbosacral eriospinal muscle.

**FIGURE 2:**
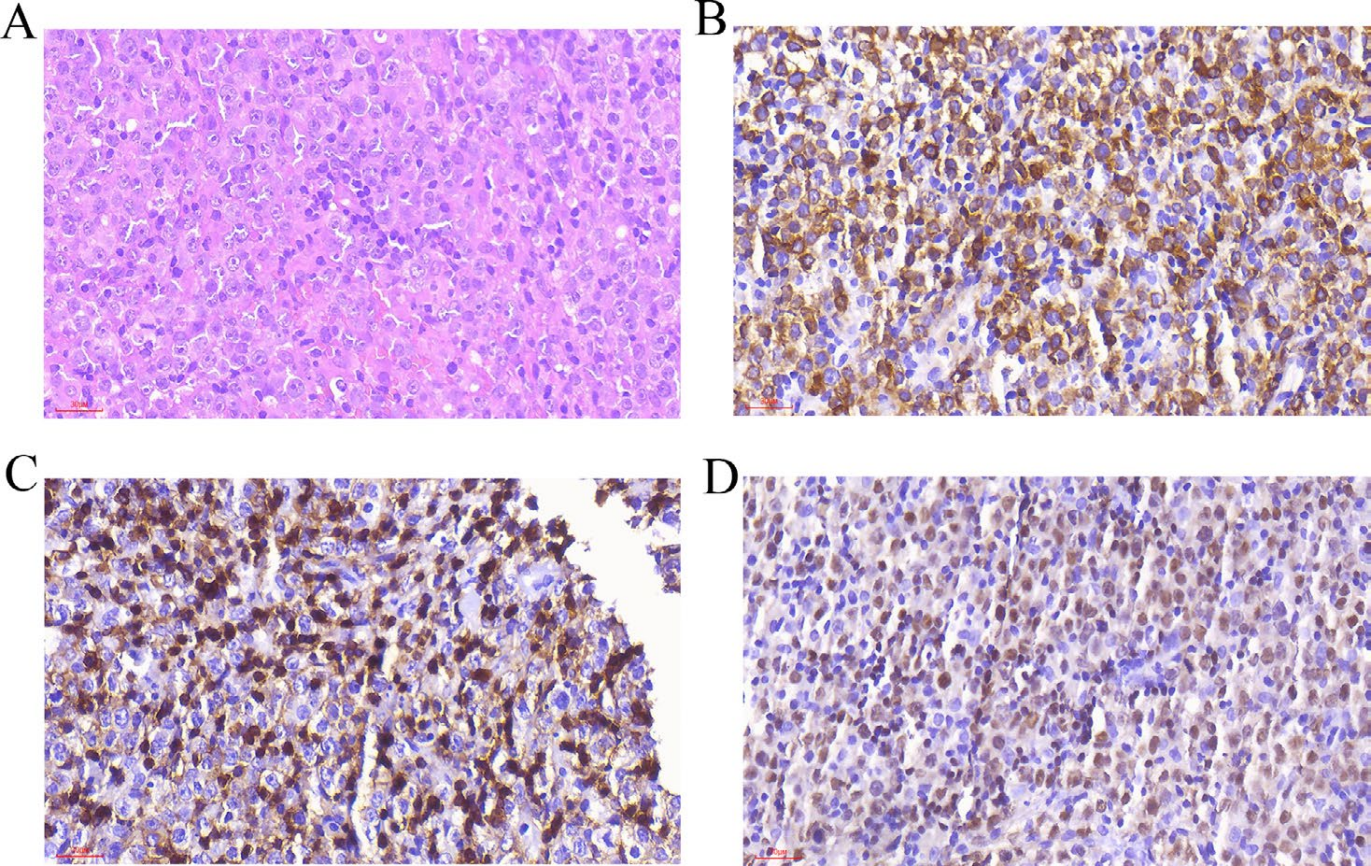
Histological examination of a left cervical lymph node. (A) Hematoxylin and eosin stain (×400) shows proliferation of medium‐sized atypical lymphocytes. Immunohistochemical analysis reveals positivity for (B) CD79a (×400), (C) CD5 (×400), and (D) PAX‐5 (×400) in the lymphocytes.

Upon admission, further laboratory investigations, chest CT, and whole‐body magnetic resonance imaging (MRI) were performed (as shown in Table 1, Figure 1). Left cervical lymph node biopsy revealed diffuse large B‐cell lymphoma, non‐germinal center B‐cell (non‐GCB) (as shown in Figure 2). Analysis of the bone marrow morphology and bone marrow biopsy did not demonstrate B‐lymphocyte proliferation. According to the revised Internal Prognostic Index (R‐IPI), patients' R‐IPI score was evaluated as 3.

**TABLE 1:** Laboratory test findings at the beginning of hospitalization.

Test	Value	Reference range
White blood cell count	1.17 × 10^9^/L	3.5–9.5
Red blood cell count	2.28 × 10^12^/L	3.8–5.1
Hemoglobin level	70 g/L	115–150
Reticulocyte count	0.045 × 10^12^/L	0.024–0.084
Reticulocyte percentage	2.28%	0.5–1.5
Platelet count	52 × 10^9^/L	125–350
Total bilirubin	57.2 μmol/L	0–20
Indirect bilirubin	49.0 μmol/L	0–17
Lactate dehydrogenase	969 U/L	109–245
Urea	5.41 mmol/L	2.86–8.2
Creatinine	27.8 μmol/L	45–84

Upon confirming the diagnosis, intravenous methylprednisolone and sodium bicarbonate were given to control hemolysis. Red blood cells and platelet transfusions were provided. After these therapeutic measures, laboratory tests showed a decrease in lactate dehydrogenase (LDH) levels. Ceftazidime was used to treat pulmonary bacterial infections. The patient commenced chemotherapy with the R‐CHOP regimen on February 8th. The patient suffered from Grade III myelosuppression and severe pneumonia, concomitant with mild shrinkage of the lymph nodes. The second course of R‐CHOP was administered on March 1st.

## 4 Outcome and Follow‐Up

**FIGURE 3:**
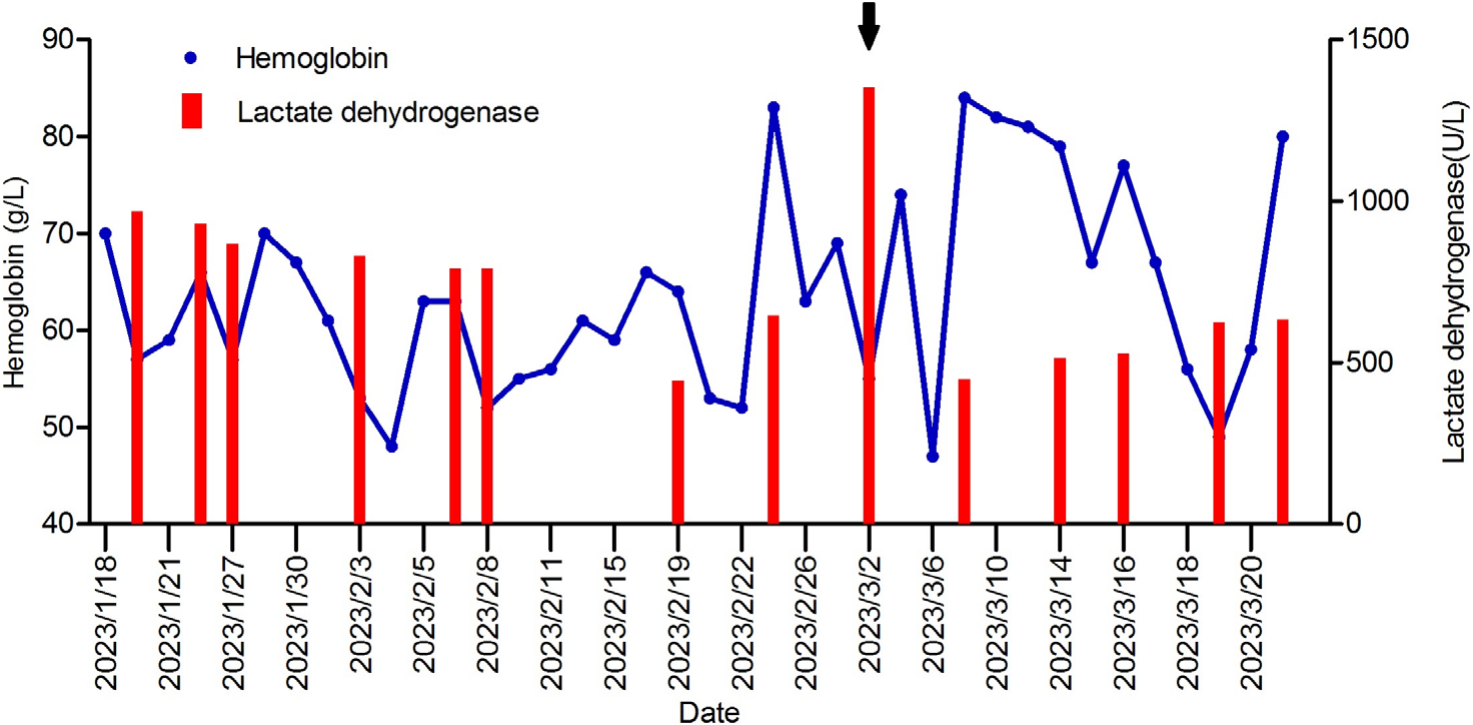
The trend graph of hemoglobin and lactate dehydrogenase (LDH) throughout the treatment course. A marked decrease in hemoglobin and a concurrent increase in LDH are observed following the second cycle of chemotherapy (arrow).

Starting from the day after chemotherapy, the serum LDH level had increased to 1353 U/L, significantly higher than pretreatment levels and indicated the development of hemolysis (as shown in Figure 3). Chest CT examination revealed worsening pulmonary infection. Over the subsequent week, an ultrasound examination revealed thrombosis in both lower limbs, and the patient developed Grade IV myelosuppression. Despite intravenous sodium bicarbonate and dexamethasone being administered, and subcutaneous low molecular weight heparin being provided, the hemolysis was not well controlled. Ultrasound examination demonstrated upper limb venous thrombosis. On March 22nd, the patient's condition rapidly deteriorated, and she succumbed to respiratory failure at last. The evidence indicates that respiratory failure was caused by a combination of pulmonary infection and pulmonary thrombosis, supported by coagulation function tests indicating fibrinogen (1.31–1.13 g/L), D‐dimer (1.47–3.21 mg/L), and ultrasound evidence of systemic multiple venous thrombosis. No autopsy was performed. Written informed consent was obtained from the patient's family members to publish this case report and any accompanying images.

## 5 Discussion

PNH is a non‐malignant clonal disorder characterized by a diverse array of clinical manifestations, including hemolytic anemia, bone marrow failure (BMF), thrombosis, as well as renal, heart, and lung failure, and several symptoms related to smooth muscle dystonia [7]. Thrombosis represents one of the most frequent and severe complications in PNH. Because of the PIG‐A mutation, PNH cells are completely deficient in GPI‐anchored proteins on their surface. Among the GPI‐APs, there are two complement‐regulatory proteins, namely CD59/MIRL (Membrane Inhibitor of Reactive Lysis) and CD55/DAF (Decay Accelerating Factor). The deficiency of such proteins leads to the increased susceptibility of PNH red blood cells (RBCs) to complement‐mediated lysis [8]. There are two classes of complement inhibitors currently used to treat PNH: C5 inhibitors—eculizumab, ravulizumab, and crovalimab—and C3 inhibitors, which include pegcetacoplan and peptide inhibitors of C3 [9, 10, 11, 12, 13, 14]. Complement inhibitors prevent intravascular hemolysis of PNH red cells by blocking the complement cascade (C3 or C5 cleavage) and inhibiting membrane attack complex formation. These inhibitors have demonstrated remarkable therapeutic efficacy, stabilizing hemoglobin levels, reducing erythrocyte transfusion requirements, decreasing thrombotic events, and improving symptom control in PNH patients [15]. Rituximab kills B cells by multiple mechanisms including complement‐dependent cytotoxicity, antibody‐dependent cellular cytotoxicity, and induction of apoptosis [16]. In DLBCL patients with concomitant PNH, pre‐treatment with complement inhibitors may blunt rituximab's complement‐dependent cytotoxicity. The indications and optimal timing for complement inhibitors in such patients require further studies. Based on this case, we suggest that patients with a high PNH clone burden (> 50%) and uncontrolled hemolysis may benefit from prophylactic complement inhibition before chemotherapy to reduce the risk of severe hemolysis and deep‐vein thrombosis during treatment.

DLBCL is the most common aggressive form of B cell lymphoma. For low‐risk DLBCL patients, the standard first‐line treatment regimen involves rituximab‐based chemoimmunotherapy such as the R‐CHOP regimen. In high‐risk patients, adding polatuzumab vedotin to the standard R‐CHOP regimen demonstrated significantly improved progression‐free survival (PFS) and reduced the need for further lines of therapy compared to the standard R‐CHOP regimen alone [17, 18]. Although the patient belonged to a high‐risk group, we ultimately chose R‐CHOP chemotherapy in consideration of lung infection and PNH clone.

Previous researches have established the presence of PNH clones in MDS and aplastic anemia (AA) [19, 20, 21, 22]. Systematic PNH testing in AA/MDS might allow better prediction/prognostication and guide the timing of consequent clinical and laboratory follow‐up [23]. Patients with PNH face a heightened risk of developing MDS and AML, with an estimated 2%–6% incidence of secondary MDS/AML within a 10‐year follow‐up period [2]. In addition to mutations in PIGA, malignancy‐associated mutations, including TET2, SUZ12, U2AF1, and JAK2, have been identified in PNH patients [24]. In PNH patients, lymphocytes had GPI deficiency and the subset of natural killer cells and B cells was found in abnormally low count. The observed alterations might suggest a compromised immune function in patients with PNH, potentially facilitating immune evasion by malignant tumor cells [25]. The occurrence of lymphoma after PNH treatment is rarely reported. Notably, no research has yet identified specific gene mutations in PNH patients that correlate with an increased risk of lymphoma.

AIHA is one complication of non‐Hodgkin lymphoma (NHL) and frequency is maximal in angioimmunoblastic T‐cell lymphoma and marginal zone lymphoma [26]. Several cases of DLBCL with AIHA have been reported and the mechanism may involve the production of inhibitory antibodies against red blood cells in lymphoma patients. The R‐CHOP regimen has effectively treated DLBCL in some patients with this co‐occurrence [6, 27, 28]. Lai et al. [5] reported a case of concurrent PNH and DLBCL who achieved long‐term remission of her PNH following chemoimmunotherapy with the R‐CHOP regimen (Table 2). However, despite receiving the same regimen, the patient we reported did not achieve remission and ultimately succumbed to pulmonary infection, hemolysis, and multiple venous thrombosis. We speculated that the following factors may have contributed to this therapeutic outcome: (1) Long‐term glucocorticoids may impair immunity and increase the risk of infection; (2) the proportion of PNH cells was higher than that reported by Sueyi et al. and PNH cells are more likely to dissolve during chemotherapy; (3) due to financial reasons, the patient did not use complement inhibitors to effectively prevent hemolysis, thereby increasing the risk of venous thrombosis; and (4) the patient's tumor burden was substantial, and the dissolution of tumor cells during chemotherapy may have precipitated thrombosis and hemolytic reactions. Despite the administration of low molecular weight heparin after chemotherapy, the patient still developed deep vein thrombosis.

**TABLE 2:** Reported cases of PNH coexisting with DLBCL: A mini‐table.

Case	Age/sex	PNH diagnosis (method)	DLBCL stage	Pre‐chemotherapy Hb (g/L)	Chemotherapy	Complement inhibitor	Thrombotic event	Outcome (follow‐up)	Citation
1	49/female	CD55/CD59 study on RBCs and WBCs	IV	81	R‐CHOP followed by radioimmunotherapy	N	N	Complete remission for lymphoma and long‐term remission for PNH	Lai et al. [5]
2	41/female	FLAER assay	IV	70	R‐CHOP	N	Y	Death	Our case

*Note:* In case 1, the PNH clone proportion was not reported and CT scan showed a large mediastinal mass, innumerable pulmonary nodules, and splenic infiltration.

Abbreviations: FLAER, fluorescently labeled inactivative aerolysin cytometry; N, no; R‐CHOP rituximab, cyclophosphamide, doxorubicin, vincristine and prednisone; Y, yes.

A case–control study has shown that, although PNH patients with a clone size > 50% and at high thrombotic risk who receive primary warfarin prophylaxis experience a significantly lower incidence of thrombosis within 10 years than those not taking warfarin (0% vs. 36.5%), major bleeding events were observed [29]. PNH patients often have concomitant thrombocytopenia, increasing bleeding risk, and the thromboembolism rate in Chinese PNH patients is markedly lower than in Western populations (6.7% vs. 32.5%) [30]. Therefore, routine prophylactic anticoagulation is currently not recommended for Chinese patients. For PNH patients with acute thrombosis and thrombocytopenia, anticoagulation should be undertaken cautiously after comprehensive bleeding‐risk assessment, with low‐molecular‐weight heparin or unfractionated heparin as first‐line agents [31]. Retrospective data indicate that the annual thromboembolic event rate during eculizumab therapy was significantly lower than before treatment (1.07% vs. 7.37%) [12]. In PNH patients with hepatic vascular involvement, eculizumab reduced mortality (2.6% vs. 8.7%) and thrombosis recurrence (0.5% vs. 2.8%) compared with the untreated period [32]. Thus, complement inhibitors should be initiated promptly after thrombosis [30].

The patient we report presented with a platelet count of 24 × 10^9^/L before the first cycle of chemotherapy and a Khorana score of 2 (intermediate risk). After chemotherapy, the platelet count fell further (10–33 × 10^9^/L), necessitating platelet transfusions. Before the second cycle, the platelet count was 18 × 10^9^/L, and the Khorana score remained 2 (intermediate risk). During chemotherapy, the patient developed acute hemolysis accompanied by bleeding from internal hemorrhoids and petechiae; the platelet count ranged from 20 to 30 × 10^9^/L, and no prophylactic anticoagulation was given. After a venous thromboembolic event occurred, anticoagulation with low‐molecular‐weight heparin sodium injection 0.4 mL (4250 IU) once daily was initiated. Although the case reported by Sueyi et al. did not experience venous thrombosis, our patient developed VTE after chemotherapy, leading us to speculate that our patient may have had a relatively higher PNH clone burden. This patient's experience suggests that, for lymphoma patients with a high PNH clone proportion (> 50%), prophylactic anticoagulation with low‐molecular‐weight heparin should be considered during chemotherapy, even in the presence of thrombocytopenia.

For our case, the optimal strategy to prevent both thrombosis and acute hemolysis would have been to initiate a complement inhibitor before chemotherapy. We explained to the family the necessity of eculizumab; however, given the patient's long disease course, complex medical history, and the substantial expenses already incurred, together with the poor curative prospects for the underlying aggressive lymphoma, the patient and family ultimately decided against eculizumab. Instead, they elected supportive care consisting of low‐molecular‐weight heparin anticoagulation, blood transfusions, and dexamethasone.

## 6 Conclusion

In contrast to the well‐documented progression of PNH to MDS/AML, secondary lymphoma development is rarely reported, and the underlying pathogenesis remains unknown. DLBCL, coexisting with PNH, exhibits distinct clinical characteristics from AIHA. In addition, factors such as pathological type, clinical stage, the proportion of PNH clone cells and treatment response of patients should be comprehensively evaluated before chemotherapy. During chemotherapy, close monitoring of routine blood parameters and liver and kidney function is essential. Additionally, monitoring LDH levels and other relevant indicators is warranted to enable timely detection and management of hemolytic crises. Furthermore, prophylactic anticoagulant therapy should be considered to mitigate the risk of thrombosis. By implementing these measures, patients may exhibit improved tolerance to chemotherapy and achieve remission. The current case report may highlight the challenges and considerations for managing DLBCL coexisting with PNH and serve as a valuable reference for clinicians.

## Author Contributions

**Zhen zhen Liu:** data curation, formal analysis, investigation, writing – original draft, writing – review and editing. **Da lin Di:** data curation, formal analysis. **An hua Feng:** data curation, formal analysis, investigation, writing – review and editing. **Jie Yu:** investigation, writing – review and editing. **Hai ying Wang:** investigation, writing – review and editing. **Lili Qin:** data curation, formal analysis, investigation, writing – review and editing.

## Ethics Statement

This case report was approved by the Institutional Review Board of the Affiliated Hospital of Shandong Second Medical University (approval number: wyfy‐2024‐qt‐023). Written informed consent was obtained from the patient for publication of this case report.

## Conflicts of Interest

The authors declare no conflicts of interest.

## Data Availability

The data presented in this study is available on request from the corresponding author.

## References

[1] R. A. Brodsky , “Paroxysmal Nocturnal Hemoglobinuria,” Blood 124, no. 30 (2014): 2804–2811, 10.1182/blood-2014-02-522128. 25237200 PMC4215311

[2] L. Sun and D. V. Babushok , “Secondary Myelodysplastic Syndrome and Leukemia in Acquired Aplastic Anemia and Paroxysmal Nocturnal Hemoglobinuria,” Blood 136, no. 1 (2020): 36–49, 10.1182/blood.2019000940. 32430502 PMC7332901

[3] R. A. Brodsky , “How I Treat Paroxysmal Nocturnal Hemoglobinuria,” Blood 137, no. 11 (2021): 1304–1309, 10.1182/blood.2019003812. 33512400 PMC7955407

[4] S. Li , K. H. Young , and L. J. Medeiros , “Diffuse Large B‐Cell Lymphoma,” Pathology 50, no. 1 (2018): 74–87, 10.1016/j.pathol.2017.09.006. 29167021

[5] S. Lai , P. Venugopal , and W. Leslie , “Long‐Term Remission of Paroxysmal Nocturnal Hemoglobinuria Following Chemoimmunotherapy for Non‐Hodgkin Lymphoma,” Clinical Advances in Hematology & Oncology 10, no. 2 (2012): 134–136. 22402359

[6] S. Kosugi , M. Watanabe , and M. Hoshikawa , “Primary Bone Marrow Lymphoma Presenting With Cold‐Type Autoimmune Hemolytic Anemia,” Indian Journal of Hematology and Blood Transfusion 30, no. Suppl 1 (2014): 271–274, 10.1007/s12288-014-0356-6. 25332595 PMC4192235

[7] U. Szlendak , B. Budziszewska , J. Spychalska , J. Drozd‐Sokołowska , E. Patkowska , and J. Nowak , “Paroxysmal Nocturnal Hemoglobinuria: Advances in the Understanding of Pathophysiology, Diagnosis, and Treatment,” Polskie Archiwum Medycyny Wewnętrznej 132, no. 6 (2022): 16271, 10.20452/pamw.16271. 35699625

[8] A. M. Risitano , D. Ricklin , Y. Huang , et al., “Peptide Inhibitors of C3 Activation as a Novel Strategy of Complement Inhibition for the Treatment of Paroxysmal Nocturnal Hemoglobinuria,” Blood 123, no. 27 (2014): 2094–2101, 10.1182/blood-2013-11-536573. 24497537 PMC3968392

[9] R. Kelly , S. Richards , P. Hillmen , and A. Hill , “The Pathophysiology of Paroxysmal Nocturnal Hemoglobinuria and Treatment With Eculizumab,” Therapeutics and Clinical Risk Management 5 (2009): 911–921, 10.2147/tcrm.s3334. 20011245 PMC2789686

[10] J. W. Lee , F. de Sicre Fontbrune , L. Wong Lee Lee , et al., “Ravulizumab (ALXN1210) vs Eculizumab in Adult Patients With PNH Naive to Complement Inhibitors: The 301 Study,” Blood 133, no. 7 (2019): 530–539, 10.1182/blood-2018-09-876136. 30510080 PMC6367644

[11] A. Röth , J. I. Nishimura , Z. Nagy , et al., “The Complement C5 Inhibitor Crovalimab in Paroxysmal Nocturnal Hemoglobinuria,” Blood 135, no. 12 (2020): 912–920, 10.1182/blood.2019003399. 31978221 PMC7082616

[12] P. Hillmen , P. Muus , U. Dührsen , et al., “Effect of the Complement Inhibitor Eculizumab on Thromboembolism in Patients With Paroxysmal Nocturnal Hemoglobinuria,” Blood 110, no. 12 (2007): 4123–4128, 10.1182/blood-2007-06-095646. 17702897

[13] A. M. Risitano and B. Rotoli , “Paroxysmal Nocturnal Hemoglobinuria: Pathophysiology, Natural History and Treatment Options in the Era of Biological Agents,” Biologics 2, no. 2 (2008): 205–222, 10.2147/btt.s1420. 19707355 PMC2721357

[14] F. Versino and B. Fattizzo , “Complement Inhibition in Paroxysmal Nocturnal Hemoglobinuria: From Biology to Therapy,” International Journal of Laboratory Hematology 46, no. Suppl 1 (2024): 43–54, 10.1111/ijlh.14281. 38622956

[15] E. Gavriilaki , R. P. de Latour , and A. M. Risitano , “Advancing Therapeutic Complement Inhibition in Hematologic Diseases: PNH and Beyond,” Blood 139, no. 25 (2022): 3571–3582, 10.1182/blood.2021012860. 34482398

[16] P. Johnson and M. Glennie , “The Mechanisms of Action of Rituximab in the Elimination of Tumor Cells,” Seminars in Oncology 30, no. 1 Suppl 2 (2003): 3–8, 10.1053/sonc.2003.50025. 12652458

[17] T. Melchardt , A. Egle , and R. Greil , “How I Treat Diffuse Large B‐Cell Lymphoma,” ESMO Open 8, no. 1 (2023): 100750, 10.1016/j.esmoop.2022.100750. 36634531 PMC9843196

[18] H. Tilly , F. Morschhauser , L. H. Sehn , et al., “Polatuzumab Vedotin in Previously Untreated Diffuse Large B‐Cell Lymphoma,” New England Journal of Medicine 27, no. 4 (2022): 351–363, 10.1056/NEJMoa2115304. PMC11702892 34904799

[19] A. Raza , F. Ravandi , A. Rastogi , et al., “A Prospective Multicenter Study of Paroxysmal Nocturnal Hemoglobinuria Cells in Patients With Bone Marrow Failure,” Cytometry. Part B, Clinical Cytometry 86, no. 3 (2014): 175–182, 10.1002/cyto.b.21139. 24227693 PMC5594745

[20] W. Wanachiwanawin , U. Siripanyaphinyo , N. Piyawattanasakul , and T. Kinoshita , “A Cohort Study of the Nature of Paroxysmal Nocturnal Hemoglobinuria Clones and PIG‐A Mutations in Patients With Aplastic Anemia,” European Journal of Haematology 76, no. 6 (2006): 502–509, 10.1111/j.0902-4441.2005.t01-1-EJH2467.x. 16529603

[21] R. E. Donohue , A. N. Marcogliese , G. S. Sasa , et al., “Standardized High‐Sensitivity Flow Cytometry Testing for Paroxysmal Nocturnal Hemoglobinuria in Children With Acquired Bone Marrow Failure Disorders: A Single Center US Study,” Cytometry. Part B, Clinical Cytometry 94, no. 4 (2018): 699–704, 10.1002/cyto.b.21536. 28574201

[22] K. Ishiyama , T. Chuhjo , H. Wang , A. Yachie , M. Omine , and S. Nakao , “Polyclonal Hematopoiesis Maintained in Patients With Bone Marrow Failure Harboring a Minor Population of Paroxysmal Nocturnal Hemoglobinuria‐Type Cells,” Blood 102, no. 4 (2003): 1211–1216, 10.1182/blood-2002-12-3706. 12676778

[23] B. Fattizzo , R. Ireland , A. Dunlop , et al., “Clinical and Prognostic Significance of Small Paroxysmal Nocturnal Hemoglobinuria Clones in Myelodysplastic Syndrome and Aplastic Anemia,” Leukemia 35, no. 11 (2021): 3223–3231, 10.1038/s41375-021-01190-9. 33664463 PMC8550969

[24] W. Shen , M. J. Clemente , N. Hosono , et al., “Deep Sequencing Reveals Stepwise Mutation Acquisition in Paroxysmal Nocturnal Hemoglobinuria,” Journal of Clinical Investigation 124, no. 10 (2014): 4529–4538, 10.1172/JCI74747. 25244093 PMC4191017

[25] J. Zhang , Z. Shen , J. Wu , and S. Jiang , “Lymphocyte Subsets Analysis and Glycosylphosphatidylinositol Determination in Patients With PNH,” Zhonghua Xue Ye Xue Za Zhi 21, no. 10 (2000): 521–523. 11877029

[26] W. Barcellini , J. A. Giannotta , and B. Fattizzo , “Autoimmune Complications in Hematologic Neoplasms,” Cancers (Basel) 13, no. 7 (2021): 1532, 10.3390/cancers13071532. 33810369 PMC8037071

[27] N. S. Ghadyalpatil , R. Chandrasekar , D. Snehalatha , and B. M. Reddy , “A Case of Primary Ovarian Lymphoma With Autoimmune Hemolytic Anemia Achieving Complete Response With Rituximab‐Based Combination Chemotherapy,” Indian Journal of Medical and Paediatric Oncology 32, no. 4 (2011): 207–210, 10.4103/0971-5851.95142. 22563154 PMC3343247

[28] H. Kuroda , T. Matsunaga , S. Iyama , et al., “De Novo CD5‐Positive Diffuse Large B‐Cell Lymphoma Associated With Autoimmune Hemolytic Anemia Presenting as Erythroid Hypoplasia,” Rinshō Ketsueki 47, no. 7 (2006): 633–638. 16910573

[29] C. Hall , S. Richards , and P. Hillmen , “Primary Prophylaxis With Warfarin Prevents Thrombosis in Paroxysmal Nocturnal Hemoglobinuria (PNH),” Blood 102, no. 10 (2003): 3587–3591, 10.1182/blood-2003-01-0009. 12893760

[30] F. Yu , Y. Du , and B. Han , “A Comparative Analysis of Clinical Characteristics of Patients With Paroxysmal Nocturnal Hemoglobinuria Between Asia and Europe/America,” International Journal of Hematology 103, no. 6 (2016): 649–654, 10.1007/s12185-016-1995-1. 27059871

[31] M. Chen , C. Yang , Z. W. Liu , et al., “Expert Consensus of Multidisciplinary Diagnosis and Treatment for Paroxysmal Nocturnal Hemoglobinuria (2024),” Medical Journal of Peking Union Medical College Hospital 15, no. 5 (2024): 1011–1028, 10.12290/xhyxzz.2024-0416.

[32] A. Plessier , M. Esposito‐Farèse , A. Baiges , et al., “Paroxysmal Nocturnal Hemoglobinuria and Vascular Liver Disease: Eculizumab Therapy Decreases Mortality and Thrombotic Complications,” American Journal of Hematology 97, no. 4 (2022): 431–439, 10.1002/ajh.26474. 35049058

